# Distyly and floral morphology of *Psychotria cephalophora* (Rubiaceae) on the oceanic Lanyu (Orchid) Island, Taiwan

**DOI:** 10.1186/s40529-015-0091-9

**Published:** 2015-05-07

**Authors:** Kenta Watanabe, T Y Aleck Yang, Chihiro Nishihara, Tai-Liang Huang, Koh Nakamura, Ching-I Peng, Takashi Sugawara

**Affiliations:** 1 Okinawa National College of Technology, 905 Henoko, Nago 905-2192 Okinawa, Japan; 2 Department of Botany, National Museum of Natural Science, Kuan Chien Rd, Taichung, 404 Taiwan; 3 Department of Life Science, National Chung Hsin University, Taichung, 402 Taiwan; 4 774-1-302, Umusa Nago, 905-0006 Okinawa, Japan; 5 Botanic Garden, Field Science Center for Northern Biosphere, Hokkaido University, North 3, West 8, Chuo-ku, Sapporo, 060-0003 Japan; 6 Biodiversity Research Center, Academia Sinica, Taipei 115, Nangang, Taiwan; 7 Makino Herbarium, Graduate School of Science, Tokyo Metropolitan University, 1-1 Minami-Ohsawa, Hachioji, Tokyo, 192-0397 Japan

**Keywords:** Breeding system, Distyly, Heterostyly, Lanyu, Oceanic island, Orchid Island, Pollen dimorphism, Psychotria cephalophora, Rubiaceae

## Abstract

**Background:**

*Psychotria cephalophora* Merr. (Rubiaceae), a shrub in oceanic islands of Taiwan and the Philippines, appears to be distylous, but distyly is usually rare on oceanic islands. To elucidate the functional breeding system of *P. cephalophora* can improve our understanding of plant reproductive ecology on oceanic islands.

**Results:**

Field investigations on Lanyu (Orchid Island) off the coast of southeastern Taiwan revealed the flowers to be distylous with short (S)- and long (L)-styled morphs, with only one morph per individual. Laboratory observations revealed that both morphs had stainable pollen grains and indicated dimorphism in stigmatic papillae and pollen size. In hand pollination experiments, the pollen tubes reached the base of the style in intermorph crossing, whereas they rarely penetrated stylar tissue in intramorph crossing and selfing. Open pollinated S- and L-styled flowers produced fruit.

**Conclusions:**

The results indicate that the breeding system of *P. cephalophora* is morphologically and functionally distylous.

**Electronic supplementary material:**

The online version of this article (doi:10.1186/s40529-015-0091-9) contains supplementary material, which is available to authorized users.

## Background

*Psychotria* L. (Rubiaceae), one of the largest genera in flowering plants, with more than 1800 species (Davis et al. [2009]), occurs mainly in tropical and subtropical regions (Hamilton [1989]; Nepokroeff et al. [1999]; Davis et al. [2001]; Sakai and Wright [2008]). Within *Psychotria*, distyly is regarded as the ancestral breeding system (Hamilton [1990]). Distyly is a genetically controlled dimorphism reported from 26 angiosperm families (Naiki [2012]). In distylous species, populations are composed of two distinct floral morphs: long (L) -styled morph with stigmas exceeding the anthers and short (S)-styled morph with anthers exceeding the stigmas. Between the two morphs male and female organs are usually at the reciprocal height (Ganders [1979]; Barrett et al. [2000]). This is a mechanism to promote animal-mediated cross-pollination.

Four species of *Psychotria*: three shrubs *P. cephalophora*, *P. manillensis*, and *P. rubra*, and a woody climber *P. serpens* are in Taiwan (Yang [1998b]). *Psychotria cephalophora* occurs only on Lanyu (Orchid Island) (Yang [1998b]) and Lutao (Green Island) off the coast of southeastern Taiwan (Chen and Lu [2008]); *P. manillensis* occurs only on Lanyu, Lutao (Yang [1998b]) and Kuei-Shan Tao (Turtle Island) off the coast of northeastern Taiwan (Tsai [2009]); *P. rubra* occurs on Taiwan Island and adjacent islands, including Lutao, but not on Lanyu; *P. serpens* occurs on Taiwan Island and adjacent islands including Lanyu. *Psychotria serpens* is distylous (Yang [1998b]; Sugawara et al. [2013]); *P. rubra* is dioecious (Yang [1998b]; Watanabe et al. [2014b]); and *P. manillensis* is monoecious (Watanabe et al. in preparation). There has been no study of the breeding system in *P. cephalophora*, except that Yang et al. ([1999]) mentioned it to be morphologically heterostylous without detailed description. The reproductive nature of *P. cephalophora* is left to be explored.

The circumscription of *Psychotria cephalophora* has been controversial. *Psychotria cephalophora* was described by Merrill ([1908]) from the Philippines, while *P. kotoensis* was described by Hayata ([1920]) from Lanyu. *Psychotria kotoensis* was later merged with *P. cephalophora* (Yang [1998b]). Recently, Sohmer and Davis ([2007]) stated, based on a study of specimens, that *P. cephalophora* from the Philippines and *P. kotoensis* from Taiwan are not conspecific, but they did not describe differences between the two species. Tao and Taylor ([2011]) provisionally applied the name *P. cephalophora* to plants from Lanyu because of the lack of data in Sohmer and Davis ([2007]). In this study, we tentatively use the name *Psychotria cephalophora* Merr. for the plants from Lanyu Island. Until now, details of the floral morphology of *P. cephalophora* have been insufficiently examined (Yang [1998b]; Sohmer and Davis [2007]; Tao and Taylor [2011]). Therefore, more information on the floral morphology of *P. cephalophora* is important in considering the taxonomy of these plants.

In this paper, we present the results of a detailed study of the floral morphology and breeding system, focusing specifically on distyly, of *Psychotria cephalophora* on Lanyu . We also briefly discuss the evolutionary significance of distyly on Lanyu Island.

## Methods

### Study site and sample collection

**Figure 1:**
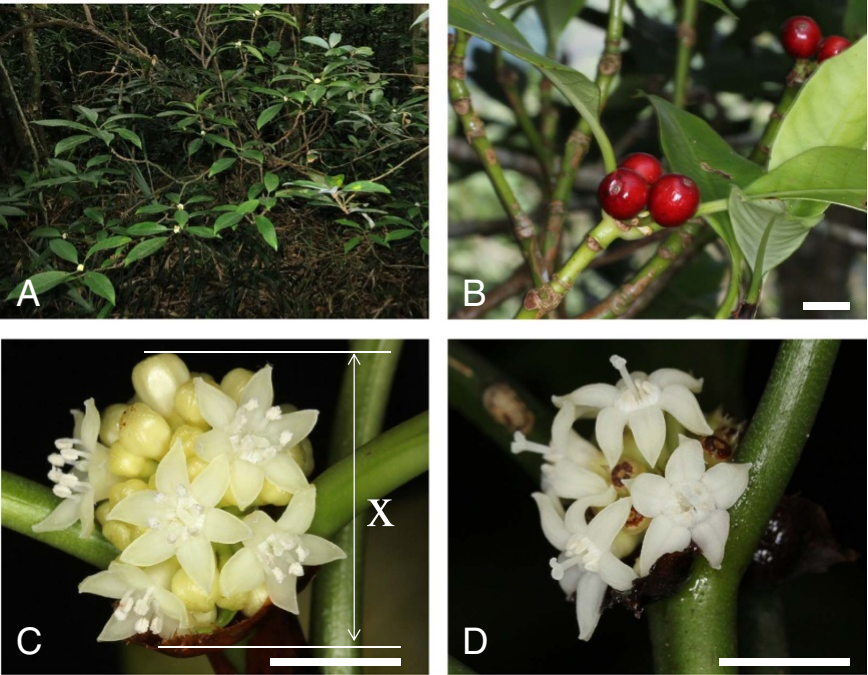
Flowers and fruit of *Psychotria cephalophora* in the Tienchi population, Lanyu Island, Taiwan. **(A)** Flowering branches. **(B)** Young fruit and inflorescences. **(C)** Short-styled morphs. **(D)** Long-styled morphs. X, diameter of inflorescence. All scale bars **(B**–**D)** = 1 cm.

*Psychotria cephalophora* on Lanyu grows to 2.5 m tall. It is terrestrial in relatively dark, wet evergreen forests (Figure 1) and blooms from February to April, according to specimen records. The fruit matures from September to January.

Observations and collections of *Psychotria cephalophora* were carried out in one natural population at Tienchi Pond (N22° 01’, E121° 34’, 337 m a.s.l.). We collected non-flowering branch cuttings on 12 September 2011 and 13 September 2012, and planted them in the greenhouse of Okinawa National College of Technology, Okinawa, Japan. To study sexual and morphological differentiation of flowers, more than two inflorescences per plant were collected from 40 plants from the Tienchi population on 30 March 2013 and preserved in 70% ethanol.

### Measurements and observations of floral morphology

**Figure 2:**
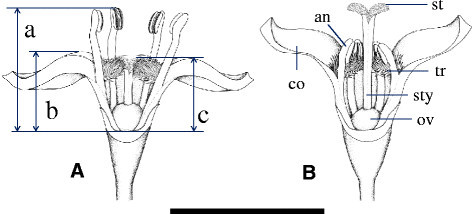
Drawings of short **(A)** and long-styled **(B)** flowers from the Tienchi population of *Psychotria cephalophora*, Lanyu Island, Taiwan. a, anther height; b, corolla height; c, stigma height. an, anther; co, corolla lobe; ov, ovary; st, stigma; sty, style; tr, oblique-facing long hairs. Scale bar = 5 mm.

Ethanol-preserved flowers from the 40 plants were used. We measured the diameter of the inflorescence (Figure 1Cx), anther height (Figure 2a), corolla height (Figure 2b), and stigma height (Figure 2c) using two flowers from different inflorescences on each plant, and the number of flowers per inflorescence was determined. The mean values of two flowers were calculated to represent each plant.

In addition to the measurements of the floral traits, we observed floral morphology of *P. cephalophora* both in the field and in the greenhouse.

### Pollen size and stainability

Equatorial and polar diameters of 30 pollen grains from a single flower were measured using a light microscope in 10 plants for each morph. We excluded pollen grains that were extremely small or had irregular shapes. Pollen stainability with aniline blue was determined using 200 pollen grains per flower for 10 plants for each morph to examine pollen viability (Kearns and Inouye [1993]). A single anther within a flower before anthesis was crushed on a glass slide with 0.05% aniline blue (Nakarai esque, Kyoto, Japan) in lactophenol, and counted the number of stained and non-stained pollen grains under light microscope. Two flowers per plant were examined to calculate the mean value.

### SEM observations of stigma and pollen

Fresh stigmas from the two morphs, collected in the greenhouse in April 2013, were observed using environmental scanning electron microscopy (SEM, S-3000 N; Hitachi, Tokyo, Japan). Pollen grains from two morphs were treated by the standard acetolysis method (Erdtman [1960]), dehydrated in an ethanol: t-butanol series, freeze-dried using a freeze-drying device (FDU-1000; Eyela, Tokyo, Japan), mounted onto SEM stubs on double sided carbon tape, coated with Pt using an ion sputter coater (E-1010; Hitachi) and observed using SEM (S-3000 N) and Field Emission SEM (S-4800; Hitachi).

### Pollination experiments

To elucidate incompatibility systems by observing pollen tube penetration in the style, intermorphic, intramorphic and self- pollinations were carried out in the greenhouse in April 2013. Approximately 24 h after hand pollination the pistils were collected, fixed in formalin acetic alcohol (FAA) solution (5 : 5 : 90 = solution of formalin : glacial acetic acid : 70% ethanol) or 70% ethanol, and transferred to new 70% ethanol for storage. The fixed styles were softened in 8 N NaOH for 24 h at 4°C, rinsed with water and then stained with 0.005% aniline blue in Na_2_HPO_4_ (pH 11) for 3 h at 4°C according to (Kearns and Inouye [1993]). The styles were mounted on a slide in glycerol beneath a cover glass and gently pressed to spread tissue. Pollen grains on the stigmas and pollen tubes in the styles were observed using a fluorescence light microscope (Axio Imager Z2; Zeiss, Oberkochen, Germany) with U-filter set 49 (Excitation 365 nm, Emission 445 nm; Zeiss).

### Statistical analyses

All statistical tests were performed using R ver. 3.0.0 software (The R Project for statistical computing; http://www.r-project.org/).

## Results

### Floral morphology and distyly

The terminal inflorescences were composed of 14 – 60 pedicellate flowers in a head-like cluster. The corolla tube, whitish and funnel shaped, averaging about 3 mm long, was glabrous outside and has white hairs in the throat. The five or six, triangular to oblong corolla lobes were about 3.5 mm long. All flowers examined had a single style, two ovules per ovary, and five or six anthers. The flowers opened in the morning and lasted until the next morning/noon. They produced a perceptible scent.

**Figure 3:**
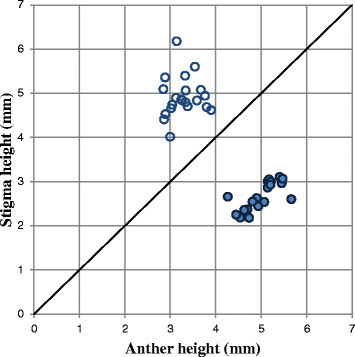
Scatter diagram showing the relationship between stigma and anther heights of *Psychotria cephalophora* in the Tienchi population. The bold line in a figure is y = x, meaning that anther and stigma heights are equal. ●, a flower of short-styled morph; ○, a flower of long-styled morph.

**Figure 4:**
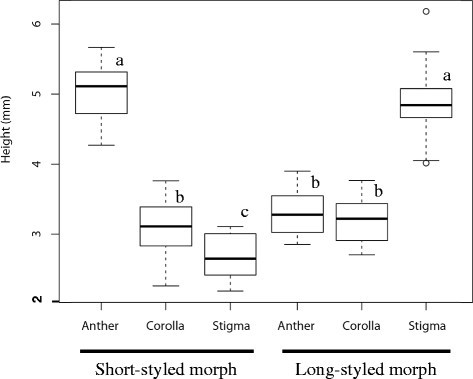
Box plots of three floral traits (anther, corolla and stigma heights) of the short- and long-styled morphs of *Psychotria cephalophora*. Small letters **(a**, **b**, **c)** indicate significant differences at *p* < 0.001 determined by Tukey-type multiple comparison after one-way ANOVA.

**Figure 5:**
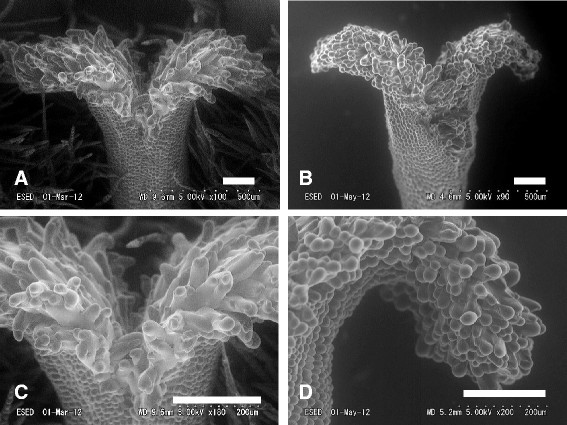
Scanning electron micrographs of stigma of short **(A**, **C)** and long-styled **(B**, **D)** morphs of *Psychotria cephalophora*. Scale bars = 200 μm.

*Psychotria cephalophora* in wild was distylous, with L- and S-styled flowers (Figure 3). Each plant had either S- or L-styled flowers. Measurements of the stigmas and anthers of the two morphs are presented in Table 1 and Figure 4. Anther height was significantly greater in the S-styled morph than in the L-styled morph, while stigma height was significantly less in the S-styled morph than in the L-styled morph (Table 1). No significant difference was found between the morphs (Table 1) in corolla length. No significant difference was found between stigma height of the L-styled morph and anther height of the S-styled morph (Figure 4). Anther height of the L-styled morph was significantly greater than stigma height of the S-styled morph but the difference in mean values was only 0.6 mm (Table 1). Anthers and stigmas of the different morphs were roughly at the same height. The stigmas were bifurcated and produced well-developed stigmatic papillae on both morphs. The fine structures of the stigmatic surface differed between the two morphs. In the S-styled morph the papillae are longer and less crowded than in the L-styled morph (Figure 5).

**Table 1:** **A comparison of several floral traits between Short- and Long-styled morphs of** ***Psychotria cephalophora***

	Short-styled morph		Long-styled morph		Mann Whitney’s
	N†	Mean ± S. D.	N†	Mean ± S. D.	***U***test
Inflorescence size (mm)	20§	18.2 ± 2.36	20	16.8 ± 1.81	ns‡
Number of flowers/inflorescence	20§	32.5 ± 7.53	20	28.1 ± 5.65	ns‡
Stigma height (mm)	20¶	2.7 ± 0.32	20	4.9 ± 0.48	p < 0.001
Anther height (mm)	20¶	5.0 ± 0.38	20	3.3 ± 0.32	p < 0.001
Corolla height (mm)	20¶	3.1 ± 0.36	20	3.2 ± 0.32	ns‡

†N: Number of individuals examined.

§:Two inflorescences on each individual were examined.

¶:Two flowers from different inflorescences on each individual were examined.

‡ns: Not significantly differnet between two floral morphs (p > 0.05).

The inflorescence was 12.9 – 23.6 mm in diameter in the S-styled morph and 11.32 – 19.99 mm in the L-styled morphs. Flowers per inflorescence ranged from 22 – 57 in the S-morph and 15 – 37 in the L-styled morph. The size of the inflorescence and the number of flowers per inflorescence were greater in the S-styled morph, but the differences between the two morphs were insignificant (Table 1).

In the Tienchi population, we found 21 L-styled morph individuals and 21 S-styled morph individuals. There was therefore no significant deviation from a 1:1 morph ratio (Binomial test: *p* > 0.05).

### Pollen stainability, size and exine sculpturing

**Figure 6:**
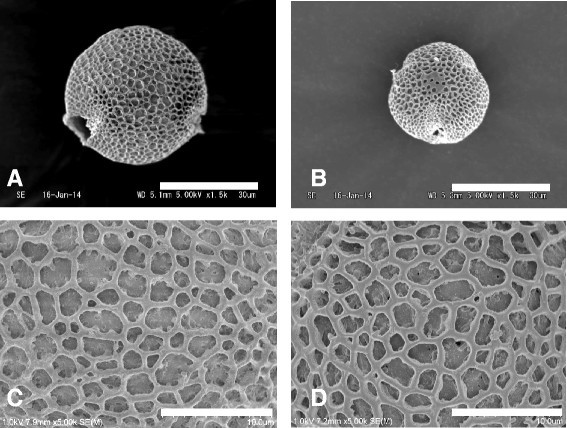
Scanning electron micrographs of pollen grains in short- **(A**, **C)** and long-styled **(B**, **D)** morphs in *Psychotria cephalophora*. Scale bars = 30 μm **(A**, **B)** and 10 μm **(C**, **D)**.

The pollen grains of *Psychotria cephalophora* were spherical, had 3 or 4 colpi, and had reticulate exine. Polar axis/ equatorial axis ratio (E/P ratio) of pollen grains was about 1.08 in both morphs. There was no significant difference between the two morphs (Mann Whitney *U*-test; *p* > 0.05). In addition, we did not notice major differences between the two morphs in exine sculpturing (Figure 6). Pollen size of the two morphs, however, was clearly different. The pollen of the S-styled morph was more than 50 μm in diameter and about 10 μm larger than in the L-styled morph (Mann Whitney *U*-test; *p* < 0.001). Pollen stainability was over 90% in both the morphs (Table 2).

**Table 2:** **Pollen size and stainability of** ***Psychotria cephalophora***

	Short-styled morph		Long-styled morph		Mann Whitney’s
	N†	Mean ± S. D.	N†	Mean ± S. D.	***U***test
Pollen size (μm)§					
Equatral	10	54.7 ± 2.5	10	44.2 ± 2.4	p < 0.001
polar	10	50.5 ± 2.7	10	41.1 ± 2.5	p < 0.001
E/P	10	1.08 ± 0.03	10	1.08 ± 0.02	ns‡
Pollen stainability (%)¶	10	93.1 ± 13.4	10	95.9 ± 2.6	ns‡

†N: Number of individuals examined.

§: 30 pollen grains from single flowers on each individual were measured.

¶: 200 pollen grains from single flowers on each individual were examined.

‡ns: Not significantly differnet between two floral morphs (p > 0.05).

### Pollination experiments

**Figure 7:**
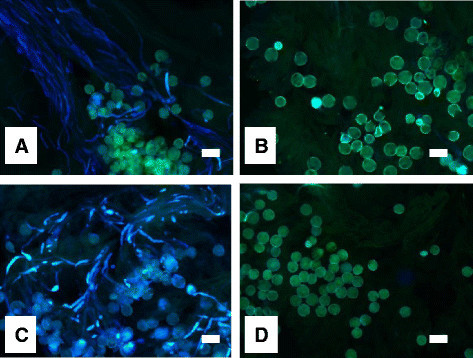
Pollen tubes in pistils approximately 24 h after intermorphic **(A**, **C)** and self- **(B**, **D)** pollinations in short- **(A**, **B)** and long-styled **(C**, **D)** morphs in *Psychotria cephalophora* observed with fluorescence microscopy. All scale bars = 100 μm.

In intermorph pollinations, more than five pollen tubes reached the base of the style in all the plants (Figure 7; Table 3). In intramorph and self-pollinated flowers, the pollen tubes did not reach the base of the style except for three cases in intramorph pollinations of the L-styled morph and two cases of selfing in the L-styled morph.

**Table 3:** **Pollen tube growth after hand pollination treatments in** ***Psychotria cephalophora***

			Status of pollen tube growth			Percentages of the pollen tube reached the base of styles
Experiments	n‡	N‡	A§	B§	C§	
Intermorphic pollination						
S → L cross†	50	7	50	0	0	100%
L → S cross†	51	8	51	0	0	100%
Intramorphic pollination						
SS cross†	30	8	0	0	30	0%
LL cross†	60	13	3	8	49	5.0%
Self pollination						
S†	77	14	0	2	75	0%
L†	61	15	2	4	45	3.3%

†S: Short-styled morph; L: Long-styled morph.

‡n: Number of flowers examined; N: Number of individuals examined.

§A: More than five pollen tubes reached at the base of the style.

B: 1 - 10 pollen tubes reached at one third of the style.

C: No pollen tube penetrate into the stigma.

## Discussion

### Distyly of *Psychotria cephalophora* on Lanyu Island

Our observations revealed *Psychotria cephalophora* to be morphologically and functionally distylous with S- and L- styled morphs. Generally, reciprocal positioning of stamens and anthers of the two morphs is characteristic of distylous species (Barrett [2002]), and reciprocal placement of the organs in distylous flowers is considered to promote disassortative pollination (Keller et al. [2014]). In *P. cephalophora*, although stigma height of the S-styled morph is significantly lower than anther height of the L-styled morph (Figure 4), the difference was only 0.6 mm (Table 1). Thus, probably this small gap does not prevent disassortative pollination between the two morphs. Although we did not observe pollinators in natural populations, moths may be one of the important pollinators because the flowers have a strong scent and white corolla (Van der Pijl [1961]). We need to study pollination in natural populations to understand the ecological significance of the positioning of the style and stamens.

The pollen of the S-styled morph is significantly larger than in the L-styled morph (Table 2), a commonly observed phenomenon in distylous species (Ganders [1979]). Darwin ([1877]) suggested that the larger pollen of the S-styled morphs is related to the need for longer pollen tubes to reach the ovules through the longer styles of the L-styled morph. However, Ganders ([1979]) found no correlation between L / S style length ratio and S / L pollen volume ratio. The adaptive significance of pollen size dimorphism in distylous plants is left to be explored. In *P. cephalophora,* the pollen exine is reticulated with no distinguishable differences between the two morphs. These features are similar in *P. serpens* (Sugawara et al. [2013]), *P. boninensis* (Kondo et al. [2007]) and *P. homalosperma* (Watanabe et al. [2014a]). Since the stamens of the two morphs produce stainable pollens they are considered to be functional. Meanwhile, both morphs have well developed stigmatic papillae, two ovules per ovary, and frequently set fruit at the study site, indicating that the flowers of both morphs of *P. cephalophora* are hermaphroditic.

In distylous plants, self- and intramorphic incompatibility is commonly observed (Klein et al. [2009]). Although we did not test fruit set, hand pollination experiments indicated that *P. cephalophora* is basically self- and intramorphic incompatible (Table 3). However, we also observed a few cases of successful self- and intramorphic pollination in the L-styled morph. This relaxed self- and intramorphic incompatibility in the L-styled morph was also reported for two distylous species of *Coussarea* and *Rudgea* (Rubiaceae) by Bawa and Beach ([1983]), although they reported that those species did not set fruit in the field. To confirm the compatibility system in *P. cephalophora* more closely, we also need to examine fruit set after hand pollination experiments. The morph ratio was approximately 1:1 in the studied population. This is also typical for distylous plant species, because distyly is a genetically controlled polymorphism following Mendelian inheritance (Barrett and Shore [2008]). We did not find any influence of partial self- and intramorphic compatibility in morph ratio.

Recently, many reproductive studies of the genus *Psychotria* have been performed in neotropics (e. g., Faivre and McDade [2001]; Castro et al. [2004]; Sakai and Wright [2008]). Among them, most species are functionally distylous, but some are intramorphically compatible, and others monomorphic. It is reported that *Psychotria carthagenesis* in Brazil shows different sexuality among different populations: typical distyly, pin-monomorphism and homostyly (Consolaro et al. [2011]; Faria et al. [2012]). Since our investigation is restricted only one population of *P. cephalophora* in Lanyu, we need to study more populations to confirm stability of its breeding system.

We described detailed floral morphology of *P. cephalophora* for the first time. To clarify whether *P. cephalophora* on Lanyu Island is endemic to the island (as *P. kotoensis*) or conspecific with the species in the Philippines, we need to examine floral traits of *P. cephalophora* in the Philippines. Molecular based analyses would also be useful to solve this issue.

### Distyly on the oceanic Lanyu Island

In this study, we reported distyly in *P. cephalophora* on the oceanic island Lanyu. Although we are unaware of the exact number of distylous species on Lanyu, there are at least several (e.g., *Ophiorrhiza japonica*, Liu [1998]; *Guettarda speciosa*, Yang [1998a]). In our preliminary observations, *P. serpens* also maintains distyly on Lanyu (Watanabe unpublished data). Distyly is, both theoretically and actually, rare or absent in oceanic islands (Carlquist [1974]; Pailler et al. [1998]; Meeus et al. [2011]; McMullen [2012]; Sugawara et al. [2014]; Watanabe et al. [2014a]). Thus it is of great interest to study how *P. cephalophora* colonized and reproduces on the oceanic island. Self-incompatible plants are thought to be less successful in colonization to oceanic islands than self-fertilizers (Baker [1955]; Stebbins [1957]; Barrett et al. [1996]); self-fertilizers can establish populations from single individual and reproduce successfully without suitable pollinators (Baker [1955]; Baker and Cox [1984]; Barrett et al. [1996]). In general, island florae have higher ratio of hermaphroditic self-compatible plants than in mainland (Crawford et al. [2011]). Lanyu and northern islands of the Philippines, where *P. cephalophora* occurs, have not been land-connected but only a few hundred km away from each other and these islands share many floristic elements (Nakamura and Kokubugata [2014]). Colonization of *P. cephalophora* into Lanyu may have occurred repeatedly unlike more isolated oceanic islands. Also Lanyu is only 60 km away from Taiwan Island and this is probably why the island has entomofauna rich enough to support self-incompatible distylous plants. There are contradictory cases where oceanic islands have a high proportion of dioecism (Carlquist [1974]; Baker and Cox [1984]; Sakai et al. [1995]; Abe [2006]). It is possibly because outcrossing has a merit to avoid inbreeding depressions in small populations on oceanic islands (Sakai et al. [1995]; Barrett et al. [1996]). Tseng et al. ([2008]) reported the proportion of dioecism on Lanyu to be very high (11.9%) compared to the global average (6%; Renner and Ricklefs [1995]) and Taiwan proper (7.9%). The outcrossing-merit hypothesis may also be applicable to distyly on Lanyu. To understand how *P. cephalophora* colonized and reproduces in natural populations, phylogeographic analyses and pollinator observations and testing open fruit sets are required.

## Conclusions

We described detailed floral morphology and incompatibility of *Psychotria cephalophora* on Lanyu for the first time. Floral dimorphism and intramorphic incompatibility indicate that the breeding system of *P. cephalophora* is morphologically and functionally distylous. Because distyly is usually rare on oceanic islands, it is important to clarify how this species colonized and reproduces on islands. Further reproductive studies of *P. cephalophora* in the natural populations on Lanyu and the Philippines are needed.

## Authors’ original submitted files for images

Below are the links to the authors’ original submitted files for images. Authors’ original file for figure 1 Authors’ original file for figure 2 Authors’ original file for figure 3 Authors’ original file for figure 4 Authors’ original file for figure 5 Authors’ original file for figure 6 Authors’ original file for figure 7
