# Supplementary figures and images for: Distyly and floral morphology of Psychotria cephalophora (Rubiaceae) on the oceanic Lanyu (Orchid) Island, Taiwan
Source: Bot Stud. 2015 May 7;56:10. doi: 10.1186/s40529-015-0091-9 (PMC5432894; doi:10.1186/s40529-015-0091-9)

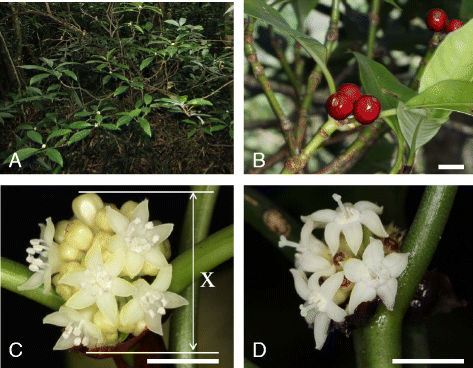

Supplement: Supplementary file 1 — Authors’ original file for figure 1 [file 40529_2015_91_MOESM1_ESM.gif]

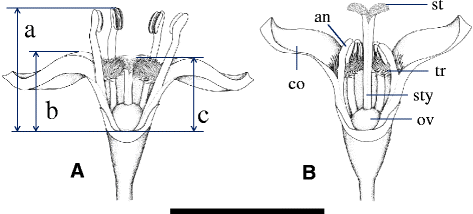

Supplement: Supplementary file 2 — Authors’ original file for figure 2 [file 40529_2015_91_MOESM2_ESM.gif]

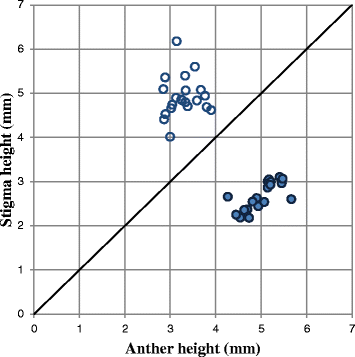

Supplement: Supplementary file 3 — Authors’ original file for figure 3 [file 40529_2015_91_MOESM3_ESM.gif]

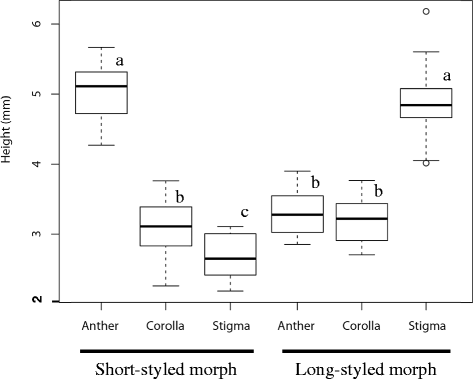

Supplement: Supplementary file 4 — Authors’ original file for figure 4 [file 40529_2015_91_MOESM4_ESM.gif]

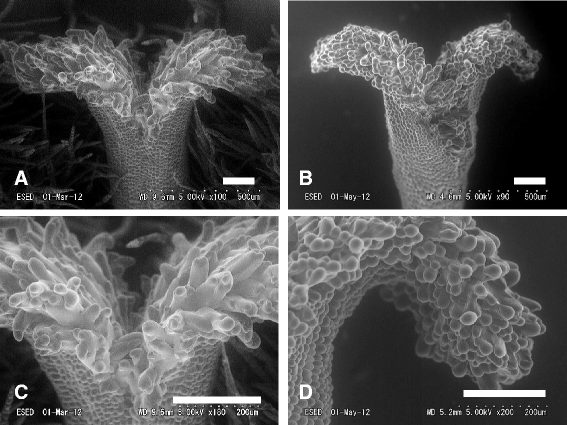

Supplement: Supplementary file 5 — Authors’ original file for figure 5 [file 40529_2015_91_MOESM5_ESM.gif]

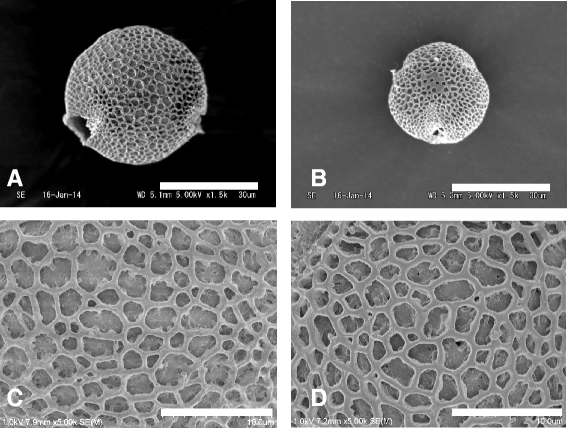

Supplement: Supplementary file 6 — Authors’ original file for figure 6 [file 40529_2015_91_MOESM6_ESM.gif]

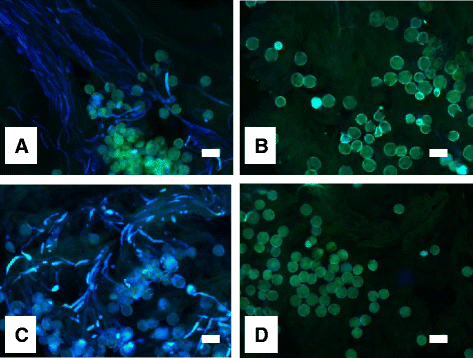

Supplement: Supplementary file 7 — Authors’ original file for figure 7 [file 40529_2015_91_MOESM7_ESM.gif]
